# Tuning Electro-Optical Characteristics through Polymerization Monomer Content in PSVA Liquid Crystal Displays: Simulation and Experimentation

**DOI:** 10.3390/polym16111606

**Published:** 2024-06-06

**Authors:** Xiaoyu Zhang, Wei Lin, Jiezhen Liu, Jiangwen Liu, Can Weng

**Affiliations:** 1 College of Mechanical and Electrical Engineering, Central South University, Changsha 410083, China; 2 Electromechanical Engineering, Guangdong University of Technology, Guangzhou 510006, China; 3 State Key Laboratory of Precision Manufacturing for Extreme Service Performance, Central South University, Changsha 410083, China

**Keywords:** polymer-stabilized vertical alignment (PSVA), polymer networks in LCDs, electro-optical performance optimization, liquid crystal mixtures

## Abstract

The enhancement of display performance and durability in polymer-stabilized vertical alignment liquid crystal and the liquid crystal are negative liquid crystals, which can be vertically aligned under the action of a vertical orientation layer and an electric field. Devices (PSVA LCDs) are crucial for advancing LCD technology. This study aims to investigate the electro-optical characteristics of PSVA LCDs by varying polymerization monomer concentrations. Using both simulations via TechWiz LCD 3D and experimental methods, such as polymer-induced phase separation, we developed an optoelectronic testing framework to assess voltage transmittance and response times. In our main findings, we show that an increase in polymeric monomer concentration from 3% to 7% resulted in a 67% increase in threshold voltage and a 44% decrease in saturation voltage. The on-state response time increased by about a factor of three, while the off-state response time decreased by about a factor of three. The alignment of our simulation results with experimental data validates our methodology, offering the potential of simulation tools as a pivotal resource in the PSVA LCDs. The alignment of our simulation results with experimental data validates our methodology, offering the potential of simulation tools as a pivotal resource in the PSVA LCDs. These advancements promise significant improvements in PSVA LCD performance and durability.

## 1. Introduction

The evolution of liquid crystal display (LCD) technology since the late 20th century has positioned it as the cornerstone of display technology for a wide array of applications, from personal devices to large-scale televisions. Among the myriad of LCD technologies, vertically aligned (VA) LCDs have emerged as a preferred choice due to their superior contrast ratios and wider viewing angles. These properties make VA LCDs ideal for high-definition televisions, computer monitors, professional displays, and even medical imaging systems where image quality is paramount. Despite these advantages, VA LCDs face challenges, including slow response times and complex manufacturing processes, a subject of extensive study in recent literature [1,2,3,4,5,6,7,8]. Addressing these challenges, recent research has focused on the integration of polymer materials with liquid crystals, aiming to enhance the stability and performance of LCDs [9,10,11,12,13,14]. Polymers can improve the alignment and stability of liquid crystals, thereby enhancing the electro-optical properties of the display. This approach has led to the development of polymer-stabilized vertical alignment (PSVA) LCDs, which offer improved response times and reduced operational voltages, making them suitable for next-generation displays that demand both high performance and reliability. Potential applications of PSVA LCDs extend beyond traditional screens to include flexible displays, augmented reality (AR) and virtual reality (VR) devices, and high-resolution automotive displays.

Incorporation of polymer networks within the liquid crystal layers has led to notable advancements in the stability and responsiveness of these displays [15,16,17,18,19,20]. Zhou et al. [21] investigated the effect of polymer network morphology on the electro-optical properties of polymer-stabilized liquid crystals and showed that the polymer network density and strength decreased with the increase of the monomer acrylate, resulting in a significant decrease in the threshold voltage and response time, which led to a decrease in the contrast ratio as well. Miao et al. [22] prepared polymer-dispersed liquid crystals with vertical orientation and explored the effects of diluent, negative liquid crystal, and Gd_2_O_3_ nanoparticle content on their electro-optical properties. The results showed that the polymer-dispersed liquid crystal devices doped with Gd_2_O_3_ nanoparticles had lower off-state transmittance and higher contrast, as well as a longer lifetime and better aging resistance. Li et al. [23] introduced bifunctional monomers (BPEFDA) and monofunctional monomers (OPPEA) with v-type polymerizable branched chains. The effects of total monomer content, different monomer ratios, and chiral dopant content on the electro-optical properties were investigated.

Researchers are also exploring new polymer materials and more efficient fabrication processes to further enhance the performance and reduce the cost of these devices. Guo et al. [24] prepared a novel thermally light transmittance controllable (TLTC) thin film using a PD&SLC system, where the transmittance can be reversibly changed from the transparent state to the light scattering state by thermal control. The film also combines the advantages of flexibility with the potential for large-scale fabrication. Li et al. [25] successfully formed polymer walls in polymer-stabilized films by UV photopolymerization and photomasking, which resulted in lower threshold voltage, significantly improved stability of electro-optical properties, reduced material cost, improved processability, and enabled the preparation of large-area flexible films. Mizusaki et al. [26] proposed a new TLTC film with polymer-stabilized vertically oriented liquid crystal devices and a novel reactive medium (RM). The intermediate (RM) and the fluorine-containing diluent (3HFFH3) showed excellent voltage-holding ratios (VHRs) upon polymerization of the light-irradiated RM. However, the current studies on the stabilized vertically oriented electro-optical properties of polymers have been carried out experimentally, and there is a lack of simulation studies on the relevant aspects. Through simulation, researchers can predict and optimize the optoelectronic properties of devices, such as contrast, brightness, viewing angle dependence, and response time, prior to actual fabrication. Simulation studies can reduce the cost of trial and error in experiments and accelerate the development process of new technologies.

This study bridges this gap by combining simulation and experimental approaches to examine the electro-optical characteristics of polymer-stabilized vertical alignment (PSVA) LCDs, with a focus on the effect of polymer monomer mass fraction. Utilizing TechWiz LCD 3D for simulation and polymer-induced phase separation for device fabrication, we aim to validate our models against empirical data, exploring the relationship between polymer content and key device parameters. Our findings reveal a significant interplay between polymer monomer concentration and device performance, including threshold and saturation voltages, as well as response times, underscoring the accuracy of our simulation approach and its implications for future LCD technology development. Prediction and optimization of the performance of PSVA liquid crystal devices can be achieved using simulation, which is important for research to improve the electro-optical performance and durability of PSVA LCDs and save time and money.

## 2. Simulation

### 2.1. Construction of the Simulation Model

**Figure 1:**
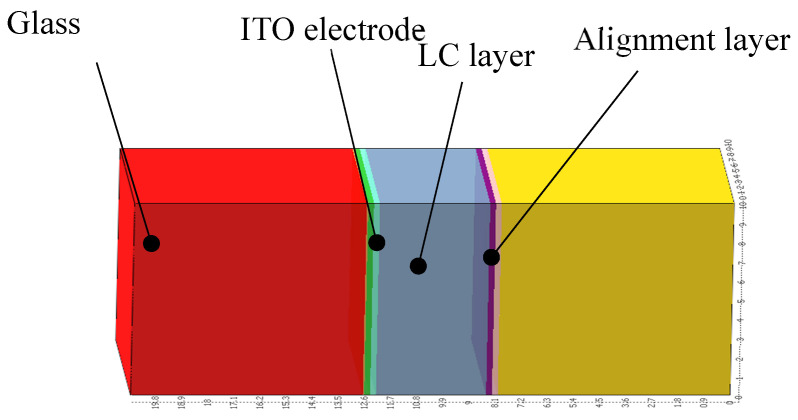
PSVA device simulation model.

In this study, we used TechWiz LCD 3D, which has a rich database, powerful 3D structural design functions, comprehensive liquid crystal dynamics and optical performance simulation, and a variety of analysis modes and simulation conditions, to develop an intricate model of a polymer-stabilized vertical alignment (PSVA) liquid crystal device, as depicted in Figure 1. Our model comprehensively represents the device architecture, including the upper and lower glass substrates, conductive layers, vertically aligned layers, and the negative liquid crystal layer. We set the number of layers of liquid crystals to 21 and the grid length to 0.5 μm. To ensure the precision of our simulations, we meticulously documented the physical properties of all materials used, as detailed in Table 1. Furthermore, Table 2 outlines the structural parameters of the model, such as the thicknesses and dimensions of each layer, ensuring a robust foundation for our simulation efforts.

### 2.2. Setting of Simulation Boundary Conditions

Our simulation workflow for PSVA liquid crystal devices is divided into two distinct phases.: the curing process and the optical simulation. During the curing phase, we precisely set the liquid crystal alignment angle to 89.5° with a fast curing time of 0.1 ms, a critical measure to achieve vertical alignment essential for PSVA device functionality. To account for the impact of varied device configurations, we adjusted the anchoring coefficients to match differing anchoring energies, mirroring the varied concentrations of polymerized monomers (3%, 5%, and 7%) in the devices. In the optical simulation phase, we meticulously established a series of specific parameters designed to accurately simulate the interaction of light with the liquid crystals. For optical simulation, the angle of the liquid crystal above the surface as a result of the curing process was used as the friction condition. Setting the distance from the glasses is 0.005μm to load the surface LC and set its pretilt angle of 89.5°. This entailed configuring light sources and polarization states, along with the application of diverse voltage settings to the model, with the applied voltage parameters elaborately detailed in Table 3.

## 3. Experimental

### 3.1. Experimental Materials

**Figure 2:**
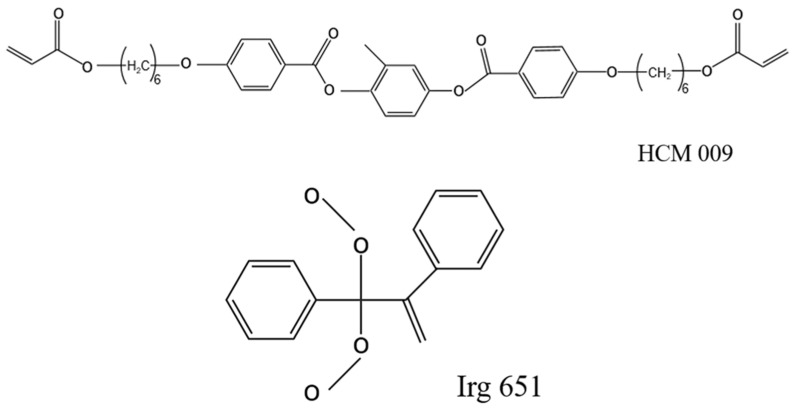
Molecular structures of HCM 009 and Irg 651 [27].

For the fabrication of polymer-stabilized vertical alignment (PSVA) liquid crystal devices, we selected specific materials for their optimal performance characteristics. The negative liquid crystals were sourced from Jiangsu Hecheng Display Technology Co., Ltd. (Nanjing, China). (HNG 30400-200), alongside the polymerization monomer (HCM 009) from the same company. The photoinitiator Irg 651 was procured from Tianjin Hynes Science and Technology Co., Ltd. (Tianjin, China), and the vertical alignment agent (DL-4018) was obtained from Shenzhen Dalton Electronic Materials Co., Ltd. (Shenzhen, China), The molecular structures of these materials are illustrated in Figure 2, and their physical properties are detailed in Table 1, ensuring a comprehensive understanding of the material basis for our experiments.

### 3.2. Preparation of PSVA Sample

The polymerizable monomers, photoinitiators, and liquid crystals are first homogeneously mixed. This mixture in the brown bottle was placed in a beaker with water and ultrasonically vibrated with the power of 80 W at 50 °C for 90 min to achieve a homogeneous blend, as detailed in Table 4. Subsequently, a glass rod was immersed in this mixture and then used to evenly disperse the mixed liquid into a liquid crystal cell, measuring 3 cm by 2 cm with a 4 μm thickness, via capillary action. The PSVA devices were then cured under a UV lamp at 40 °C for 5 min with a UV curing intensity of 10 mW/cm^2^ and subsequently allowed to cool to room temperature. This process was replicated to prepare devices with varying polymer monomer mass ratios of 3% (Sample A), 5% (Sample B), and 7% (Sample C), enabling a comparative analysis of the effects of polymer concentration on the device’s performance.

### 3.3. Characterization

**Figure 3:**
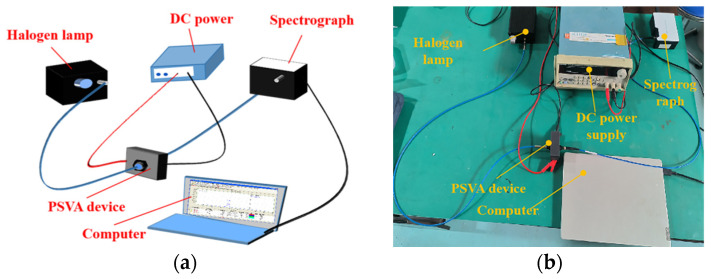
PSVA device electro-optical characteristics of the test optical path schematic and physical drawing: (**a**) schematic; (**b**) physical drawing.

The electro-optical characteristics of the PSVA devices were evaluated using a detailed optical test setup, as depicted in Figure 3. A halogen light source (Hangzhou Saiman Technology Co., H03, Hangzhou, China) directed incident light through an optical fiber onto the PSVA device. Variable DC voltages were applied at both ends of the device to modulate the light transmission, which was then captured through an optical fiber by a spectrometer (Hangzhou Saiman Technology Co., S200-Vis, Hangzhou, China). The transmitted light data were processed using Spectra V3.4 software to generate voltage transmittance curves for each sample.

**Figure 4:**
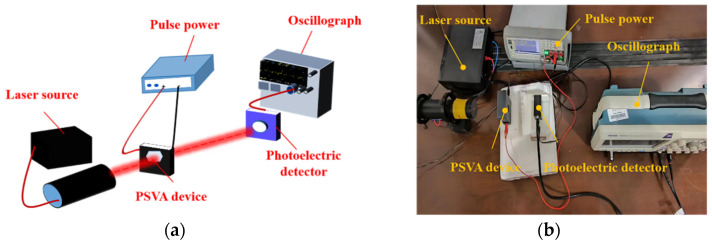
PSVA device response time test optical path schematic and physical drawing: (**a**) schematic; (**b**) physical drawing.

Additionally, the response time of the PSVA devices was assessed using a setup illustrated in Figure 4. A laser light source (KEWLAB HNR, 632.8 nm, Hangzhou, China) illuminated the sample, with a pulsed power supply (Shenzhen RongDaXin, RDX-DP) applying a square wave voltage signal with a 500 ms period to the device. Optical signals received by a photodetector (Beijing Hamamatsu Photonics Technology Co., Ltd., Beijing, China) were converted into electrical signals, which were then analyzed by an oscilloscope to derive response time curves.

The morphology of the polymer within the PSVA devices was meticulously investigated using scanning electron microscopy (SEM, Czech TESCAN Brno,s,r,o, Helios 5 CX), whose detector is secondary electrons and acceleration voltage between 200 V and 30 KV is continuously adjustable. For SEM observation, the samples were first split and immersed in cyclohexane for 30 days, with periodic replacement of the solvent to ensure complete removal of liquid crystal molecules. The samples were then dried in a vacuum oven at 40 °C for 24 h. A final treatment involved coating the samples with a thin layer of gold to enhance conductivity, preparing them for detailed SEM analysis.

## 4. Results and Discussion

### 4.1. Analysis of PSVA Device Optoelectronic Performance Simulation Results

**Figure 5:**
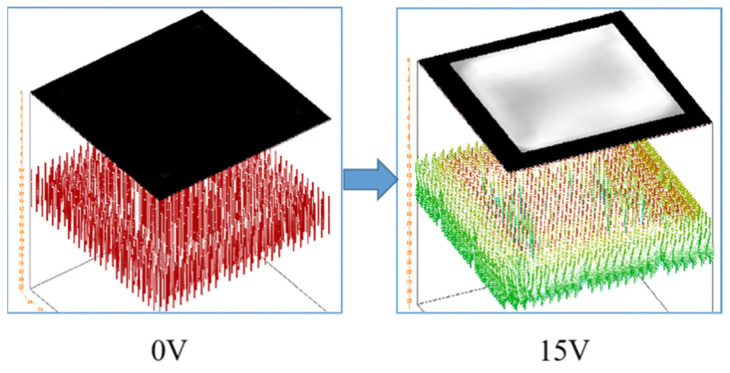
Simulation of PSVA device transmittance variation.

**Figure 6:**
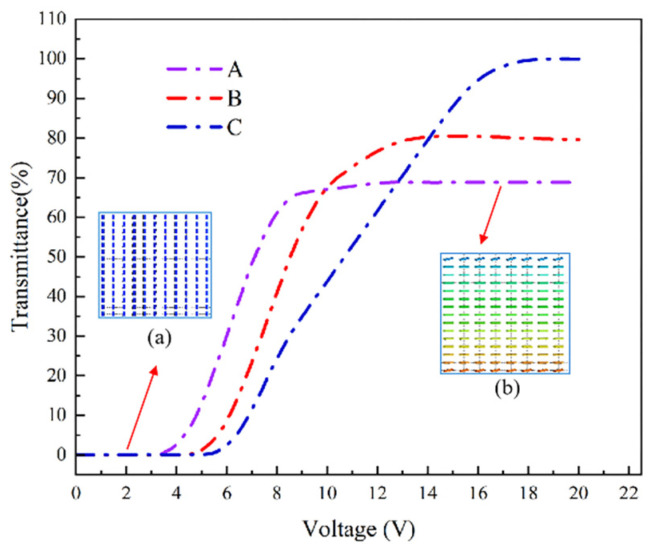
Voltage transmittance curves of samples A, B, and C in the simulation. (**a**) Liquid crystal molecules aligned vertically on the substrate. (**b**) Liquid crystal molecules arranged in parallel substrate.

Our simulation analysis, as depicted in Figure 5, unveiled distinctive transmittance behaviors of polymer-stabilized vertical alignment (PSVA) devices under various voltage conditions. At 0 V, the liquid crystal molecules are aligned perpendicularly to the substrates, facilitated by a vertical orienting agent, resulting in a ‘normally black’ mode due to the orthogonal arrangement of polarizers on either side of the substrate (Figure 6a). In this state, light passing through the first polarizer has its polarization direction perpendicular to the second polarizer, effectively blocking the light and rendering the liquid crystal display (LCD) dark. Upon applying a saturation voltage of 15 V, a significant shift occurs in the orientation of the liquid crystal molecules. They transition from their initial vertical alignment to a parallel configuration relative to the substrates, as illustrated in Figure 6b. This reorientation allows the liquid crystal molecules to alter the polarization state of the traversing light, enabling it to pass through the orthogonally positioned polarizers, thereby turning the LCD transparent.

**Figure 7:**
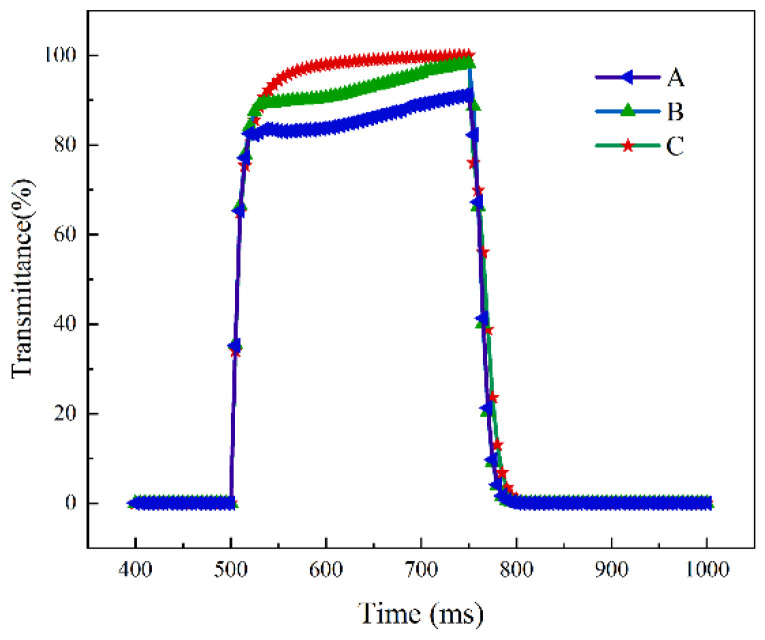
Response time curves for samples A, B, and C in the simulation.

The relationship between voltage and transmittance for samples A, B, and C, detailed in Figure 6, underscores a gradual increase in transmittance with voltage, peaking at higher voltages. Notably, threshold voltages for these samples were observed at 4.8 V, 6.4 V, and 7.5 V, respectively, indicating an upward trend in threshold and saturation voltages (9.3 V, 11 V, and 14.6 V, respectively) as polymerization monomer content increases. Further examination, as shown in Figure 7, presents the response time curves for these PSVA devices. The on-state response time (measured from the transmittance increase from 10% to 90% following voltage application) exhibits a decrease across the samples: 13.14 ms for Sample A, 22.63 ms for Sample B, and 33.74 ms for Sample C. Conversely, the off-state response time, which tracks the transmittance decrease from 90% to 10% post-voltage removal, shows a progressive increase, with times recorded at 25.12 ms, 19.7 ms, and 8.8 ms, respectively.

### 4.2. Analysis of Experimental Results on the Photovoltaic Performance of PSVA Devices

**Figure 8:**
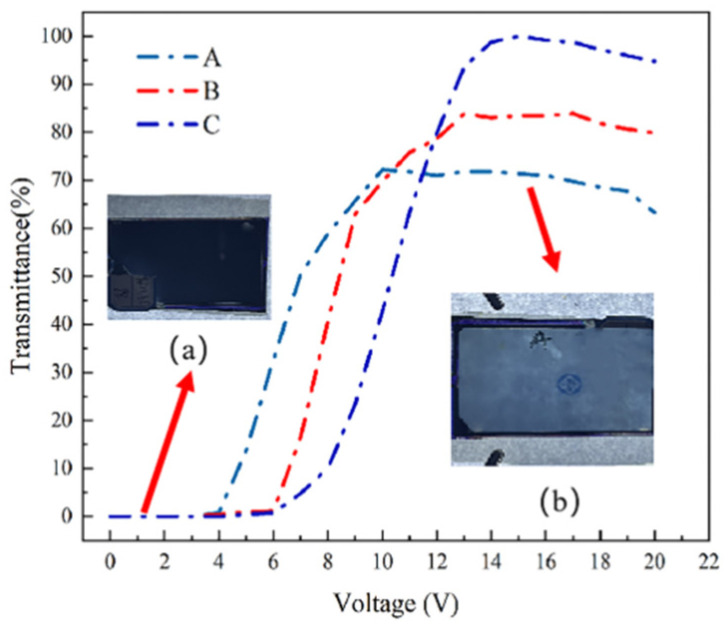
Voltage transmittance curves of PSVA devices with samples A, B, and C in the experiment. (**a**) PSVA devices in the absence of voltage; (**b**) PSVA devices at saturation voltage.

Our experimental investigation into PSVA devices, encompassing varying polymerization mass fractions, unveiled significant trends in voltage transmittance behaviors, as delineated in Figure 8. Across samples A, B, and C, we observed a consistent, gradual increase in transmittance from zero, peaking at elevated voltage levels. As in Figure 8a,b, the PSVA device is in an all-black state with the transmittance at zero, and the school logo pattern at the bottom cannot be seen. As the voltage increases, the transmittance rate gradually increases, and the school logo pattern appears clearly. This uniform behavior showcases distinct nuances as the polymerization mass fraction varies. Notably, threshold voltages for these samples were recorded at 4.3 V, 6.2 V, and 7.2 V, respectively, with saturation voltages observed at 9 V, 10.5 V, and 13 V. These findings indicate an incremental rise in both threshold and saturation voltages concurrent with the increase in polymerization monomer content.

**Figure 9:**
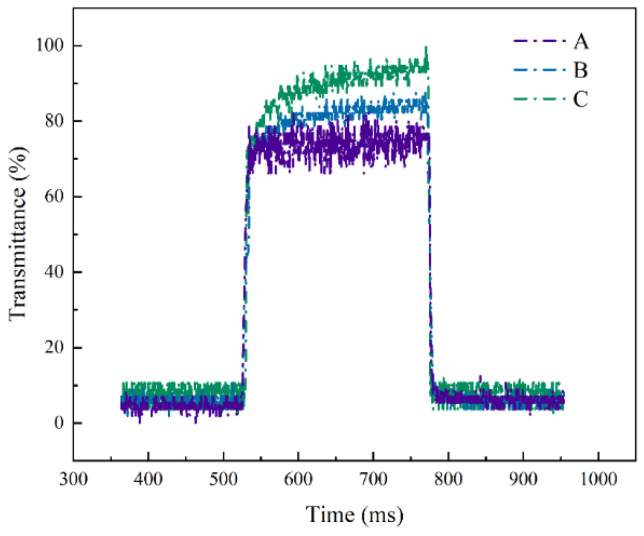
Response time curves for PSVA devices with samples A, B, and C in the experiment.

Moreover, the response time dynamics over a single cycle for samples A, B, and C, as illustrated in Figure 9, underline the impact of polymerization monomer mass fraction on device performance. An increase in the monomer mass fraction correlates with elongated on-state response times recorded at 11.6 ms for Sample A, 21 ms for Sample B, and 30.8 ms for Sample C, attributable to the denser polymer network’s augmented anchoring force. Conversely, off-state response times of 25.2 ms, 14 ms, and 8.4 ms for Samples A, B, and C, respectively, demonstrate a decline, highlighting the rapid realignment of liquid crystals upon voltage removal due to the stronger anchoring energy of devices with higher monomer mass fractions.

**Figure 10:**
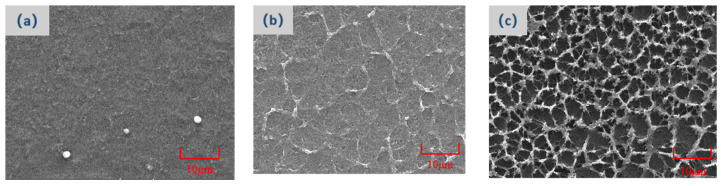
The polymer morphologies formed by different polymerization monomer contents. (**a**) Sample A; (**b**) Sample B; (**c**) Sample C.

The mechanism underlying this trend is the densification of the polymer network with increased monomer concentration. Figure 10 elucidates the escalating network density alongside the rising polymerization monomer concentration. At the lowest examined concentration (3%), the polymer network is scarcely visible (Figure 10a), a manifestation of inadequate monomer content for effective cross-linking and dense network formation. As the concentration escalates to 5%, observable cross-linking occurs, albeit resulting in a sparser network (Figure 10b). Elevating the concentration to 7% markedly increases the network’s compactness (Figure 10c), enhancing the anchoring effect on liquid crystal molecules and necessitating higher voltages for molecular deflection. As the polymerization monomer content increases, a consequent enhancement in the density of the polymer network is observed. This densification results in an augmented anchoring force, which must be overcome for the deflection of liquid crystal molecules. Consequently, a higher voltage is required to surpass this increased anchoring force, leading to elevated threshold and saturation voltages. This dynamic also influences the electro-optical response times of the devices. Specifically, the on-state response time, reflecting the duration needed to overcome the anchoring force, is prolonged. Conversely, upon the withdrawal of the voltage, the pronounced anchoring force facilitates a swifter reorientation of the liquid crystal molecules to their original alignment, thereby reducing the off-state response time.

These experimental insights underscore the critical role of polymerization monomer concentration in modulating the dynamic behavior of liquid crystal molecules within PSVA devices, offering profound implications for the design and optimization of these devices towards enhanced performance.

### 4.3. Comparative Analysis of Experimental and Simulation Results on the Electro-Optical Performance of PSVA Devices

**Figure 11:**
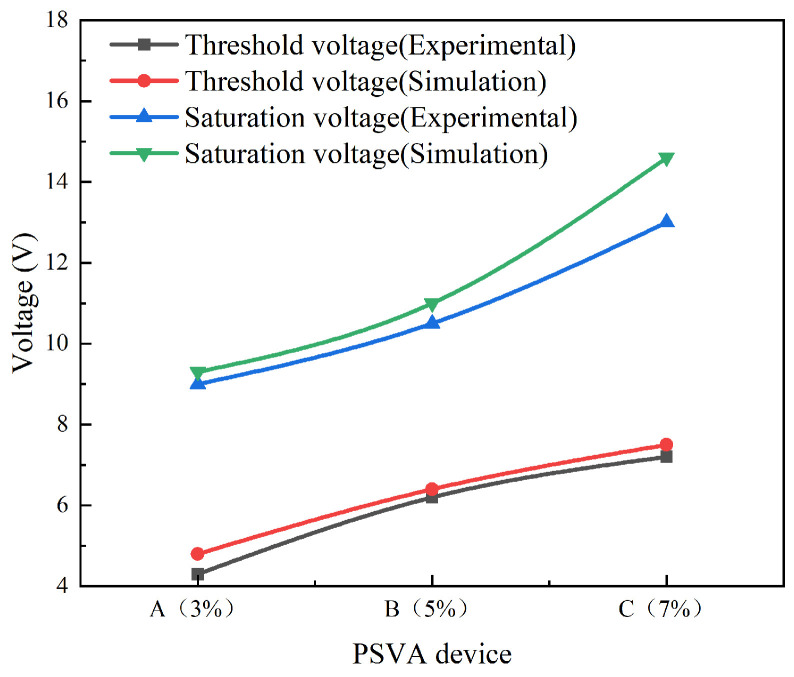
Comparison of simulated and experimental electro-optical characteristics of samples A, B, and C.

**Figure 12:**
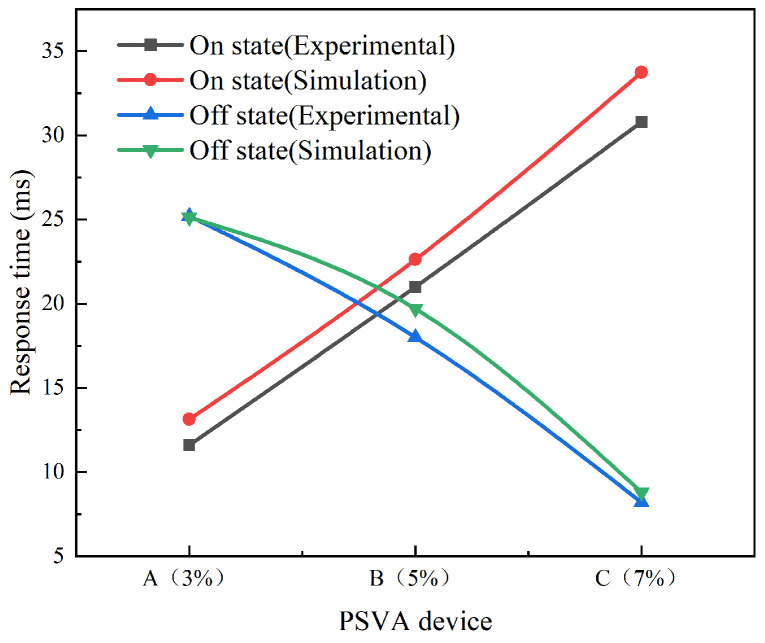
Comparison of simulated and experimental response characteristics of samples A, B, and C.

The comparative analysis, depicted in Figure 11 and Figure 12, scrutinizes the electro-optical performance of PSVA devices, juxtaposing simulation outcomes with experimental data. This analysis elucidates that PSVA devices predominantly operate in a ‘normally black’ mode under zero voltage. Upon voltage application, we observe a consistent and gradual increase in transmittance, peaking at the saturation voltage before stabilizing. This phenomenon aligns with expectations for PSVA device behavior, showcasing their electro-optical versatility.

A uniform trend across different polymerization monomer concentrations was noted: escalating monomer content is directly proportional to increases in threshold and saturation voltages. This observation is coupled with a notable rise in open-state response times and a reduction in off-state response times, indicating a nuanced interplay between polymer content and device responsiveness. The striking concordance between experimental results and simulation predictions underscores the robustness and reliability of our simulation methodology, affirming its value as a predictive tool in PSVA device development.

However, a critical observation reveals that simulation outcomes generally register slightly higher than their experimental counterparts, exhibiting a deviation of up to 12%. In our research simulation and experiment, the simulation results can accurately reflect the trend of the experimental data, but the numerical difference increases. The source of error is mainly in the experimental process, in the experimental process, the uniformity of the liquid crystal and polymer, the degree of light avoidance in the process of filling the crystal, etc., will affect the polymer polymerization to form the weaving process, which affects the optoelectronic properties. And the simulation is in an ideal state, so there is a deviation. The slight deviations observed prompt a deeper investigation into the dynamic complexities of PSVA devices, suggesting areas for further refinement in both simulation approaches and experimental methodologies. This nuanced understanding of the correlation and discrepancies between simulated and real-world behaviors is instrumental in advancing PSVA technology, driving towards more accurate predictive models and optimized device performance.

## 5. Conclusions

This study leveraged the capabilities of Tech Wiz LCD 3D v15.0 simulation software to investigate the electro-optical performance of polymer-stabilized vertical alignment (PSVA) liquid crystal devices, focusing on the effects of varying polymerized monomer concentrations. Through meticulous fabrication and the use of polymer-induced phase separation, PSVA devices with monomer contents of 3%, 5%, and 7% were produced. The establishment of an optoelectronic performance testing platform facilitated the acquisition of critical data, including voltage transmittance and response time curves, providing a comprehensive empirical basis for our analysis.

Our findings indicate that increases in polymerized monomer content lead to significant rises in both threshold and saturation voltages, with devices containing 7% monomer content demonstrating optimum performance within the low voltage range and maintaining rapid response times. This highlights the devices’ capability for efficient operation and underscores the critical influence of polymer content on their electro-optical properties.

The primary novelty of our work lies in the detailed correlation between polymer content and device performance using the methods of simulation and experiment. This nuanced understanding provides a clearer pathway for fine-tuning PSVA devices for specific applications, surpassing prior studies that did not establish such explicit performance thresholds. Moreover, the congruence between simulated and experimental results not only validates our simulation model but also highlights the potential of simulation tools as a pivotal resource in the field. These tools offer promising avenues for the development of innovative polymer formulations and structural designs, aiming to enhance device performance and cost-efficiency.

Ultimately, this research contributes significantly to the understanding of PSVA liquid crystal devices, showcasing the utility of simulation in advancing display technology. Our insights into optimizing device performance, alongside the potential for cost reduction, underscore the evolving landscape of liquid crystal display technology and its ongoing development.

## Figures and Tables

**Table 1:** Some physical properties of the materials used.

Material	Type	Parameters
Negative liquid crystal (VA-LC)	HNG 30400-200	TN1 = 94 °C Δε = −8.3 Δn = 0.224
		Viscosity[mm^2^·s^−1^, 25 °C] = 44
		K11[pN] = 16.7 K22[pN] = 7.3 K33[pN] = 18.1
		Pitch[μm]= INFINITE
Polymeric monomer	HCM 009	252 nm (Peak of absorption)
		337 nm
Photoinitiator	Irg 651	
Vertical alignment agent (PI)	DL-4018	$\varepsilon_{r}$ = 3.8
Glass		$\varepsilon_{r}$ = 4.5

**Table 2:** PSVA device structure parameters.

Layers	Thicknesses (μm)	Long × Width (mm × mm)
Glass substrates	10	20 × 20
ITO electrode	0.1	16 × 16
Vertical alignment layer	0.1	16 × 16
LC layer	4	16 × 16

**Table 3:** Parameters of voltage at both ends of electrodes.

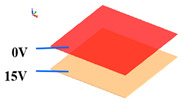	Voltage Value
Scan voltage	[0, 1, 20]
Signal voltage	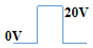

**Table 4:** Compositions of the samples studied.

Sample	HNG 30400-200/HCM 009/Irg 651 (wt%)
A	96.8/3/0.2
B	94.8/5/0.2
C	92.8/7/0.2

## Data Availability

Data are contained within the article.
